# Exogenous Ganglioside GM3 Attenuates Atherosclerosis via Multi-Organ Modulation of Lipid Metabolism

**DOI:** 10.3390/pharmaceutics18050547

**Published:** 2026-04-29

**Authors:** Jinhua Zhou, Hongda Zhuang, Qinghua Sheng, Zhitao Qiu, Yong Chen

**Affiliations:** 1 Institute for Advanced Study, Nanchang University, Nanchang 330031, Chinahongdadazhuang@gmail.com (H.Z.);; 2 The MOE Basic Research and Innovation Center for the Targeted Therapeutics of Solid Tumors, School of Pharmacy, Nanchang University, Nanchang 330031, China; 405600240069@email.ncu.edu.cn; 3 College of Life Sciences, Nanchang University, Nanchang 330031, China

**Keywords:** atherosclerosis, Ganglioside GM3, lipid metabolism, VLDL secretion, cholesterol absorption

## Abstract

**Background:** Atherosclerosis (AS) remains a leading cause of cardiovascular mortality worldwide and is significantly driven by hyperlipidemia. While ganglioside GM3 is known to regulate cellular lipid metabolism, its systemic pharmacological effects on atherosclerosis remain unclear. This study aims to evaluate the anti-atherosclerotic efficacy of exogenous GM3 and elucidate its underlying systemic mechanisms. **Methods:** C57BL/6N ApoE^−/−^ mice fed a high-fat diet were intravenously treated with exogenous GM3 (1 or 4 mg/kg) every three days for 12 weeks. Atherosclerotic progression and lipid profiles were evaluated through histological analyses of the aortic arch and aortic sinus, alongside biochemical and molecular assessments of plasma, hepatic, and intestinal tissues. **Results:** GM3 treatment significantly reduced plaque formation in the aortic arch and aortic sinus, along with decreased plasma levels of triglycerides, total cholesterol, and LDL-C. Mechanistically, GM3 suppressed hepatic VLDL secretion by downregulating ApoB100 and MTTP expression. Concurrently, hepatic lipid clearance was enhanced via the upregulation of *Ldlr*, *Scarb1*, and *Lrp1*. GM3 also lowered circulating PCSK9 levels and reduced intestinal cholesterol absorption by decreasing NPC1L1 expression. Although GM3 promoted lipid accumulation in the liver, no evidence of liver dysfunction or systemic toxicity was observed. **Conclusions:** Exogenous GM3 acts as a potent multi-target modulator that attenuates atherosclerosis by coordinating hepatic lipoprotein metabolism and restricting intestinal cholesterol uptake. This multi-organ metabolic partitioning strategy highlights the potential of GM3-based therapeutics for managing complex dyslipidemia.

## 1. Introduction

Atherosclerosis (AS) serves as a common pathological basis for various life-threatening vascular disorders, including coronary heart disease, stroke, and abdominal aortic aneurysm (AAA) [1]. It remains the leading cause of cardiovascular mortality worldwide, manifesting clinically as myocardial infarction and stroke [2,3]. Among the established risk factors, elevated low-density lipoprotein cholesterol (LDL-C) stands as the primary driver of plaque initiation and progression, triggering the endothelial activation that precedes lesion formation [4].

While lipid-lowering therapies remain the cornerstone of cardiovascular management, the persistence of ‘residual cardiovascular risk’ underscores the necessity of novel strategies that target lipid metabolism through alternative mechanisms [5,6]. Gangliosides are bioactive glycosphingolipids that play pivotal roles in diverse physiological and pathological processes, ranging from cancer progression to cardiovascular regulation [7]. GM3 is a monosialodihexosylganglioside, characterized by the presence of a sialic acid (neuraminic acid) residue linked to a ceramide lipid anchor and an oligosaccharide chain. Within this family, Ganglioside GM3 presents a fascinating biological paradox: it accumulates significantly in advanced atherosclerotic lesions—correlating with severity [8]. However, our recent investigations suggest this may not be a pathological driver, but rather an adaptive, albeit insufficient, host defense mechanism aimed at limiting vascular injury [9]. This raises a compelling question: if we amplify this endogenous response through exogenous administration, can we tip the balance from localized accumulation to systemic protection? Extending our focus beyond the vessel wall, we hypothesized that the therapeutic potential of GM3 involves a coordinated regulation of systemic lipid flux.

Our previous investigations support the protective response hypothesis. We demonstrated that exogenous GM3 structurally stabilizes LDL particles to prevent oxidation and inhibits monocyte adhesion to the endothelium, effectively targeting the local inflammatory components of atherogenesis [10]. We also observed that GM3 administration significantly lowers circulating lipid levels in vivo, a systemic effect that cannot be fully explained by local vascular mechanisms.

In this study, we hypothesize that the anti-atherosclerotic efficacy of exogenous GM3 extends beyond the vessel wall and involves a coordinated regulation of systemic lipid metabolism. Moving beyond local vascular effects, we aimed to elucidate how GM3 influences the “source” and “clearance” of circulating lipids. Specifically, we investigated its regulatory roles in hepatic lipoprotein assembly, receptor-mediated clearance, and intestinal cholesterol absorption, providing a comprehensive mechanistic framework for its therapeutic potential in dyslipidemia.

## 2. Materials and Methods

### 2.1. Materials and Chemicals

Ganglioside GM3 sodium salt (Cat. No. AG-CN2-9002-M001; AdipoGen Life Sciences, San Diego, CA, USA) with a purity of ≥98% was used in this study. According to the manufacturer’s analytical specifications, this GM3 was isolated from bovine brain. Its ceramide lipid anchor consists of a sphingosine base (C18:1-C20:1) and is predominantly composed of stearic acid (C18:0) at a proportion of over 90%. High-fat diet (HFD) containing 21% fat and 0.15% cholesterol was obtained from Hunan SJA Laboratory Animal Co., Ltd. (Changsha, China). For biochemical assays: Diagnostic kits for total cholesterol (TC), triglycerides (TG), low-density lipoprotein cholesterol (LDL-C), and high-density lipoprotein cholesterol (HDL-C) were purchased from Anhui Ipnocon Biotechnology Co., Ltd. (Hefei, China). Assay kits for alanine aminotransferase (ALT) and aspartate aminotransferase (AST) were obtained from Nanjing Jiancheng Bioengineering Institute (Nanjing, China). Antibodies: Antibodies against ApoB100, MTTP, HMGCR, DGAT1, and β-Actin were obtained from Affinity Biosciences (Cincinnati, OH, USA).

### 2.2. Animals and Treatment

C57BL/6N Male ApoE^−/−^ mice (8 weeks old) were purchased from Hunan SJA Laboratory Animal Co., Ltd. (Hunan, China). All animal procedures were approved by the Ethics Committee of Jiangxi University of Chinese Medicine. After one week of acclimation, the mice were randomly assigned to four groups (*n* = 6 per group): (1) Control group (standard chow); (2) HFD group (high-fat diet, HFD, containing 21% fat and 0.15% cholesterol was obtained from Hunan SJA Laboratory Animal Co., Ltd. (Changsha, China); (3) HFD + GM3^low^ group (HFD + 1 mg/kg GM3); and (4) HFD + GM3^high^ group (HFD + 4 mg/kg GM3). Ganglioside GM3 (AG-CN2-9002-M001, AdipoGen Life Sciences) was dissolved in phosphate-buffered saline (PBS). For the animal experiments, mice in both the Control and HFD groups were administered an equal volume of PBS, which effectively served as the vehicle control. GM3 or PBS was administered via tail vein injection every three days for 12 weeks. Body weight was monitored weekly. At the end of the 12-week experimental period, the mice were fasted overnight and euthanized via isoflurane anesthesia. Blood samples were collected, and the mice were perfused with PBS. Target organs, including the entire aorta, heart, and liver, were harvested for subsequent analyses.

### 2.3. Plasma Biochemical Analysis

Blood samples were collected via retro-orbital sinus puncture into lithium heparin anticoagulant tubes after a 12 h overnight fast. The collected blood was allowed to stand for 30 min at room temperature, and then plasma was isolated by centrifugation at 3000 rpm for 10 min at 4 °C for further biochemical analysis.

Lipid Profiles: Plasma concentrations of TC, TG, LDL-C, and HDL-C were quantified using an automated biochemical analyzer (Beckman Coulter AU480, Beckman Coulter Inc., Brea, CA, USA) with their respective enzymatic kits.

Liver Function Tests: Plasma ALT and AST activities were determined using a microplate-based colorimetric method. The assays were performed according to the manufacturer’s instructions, and absorbance was measured at 510 nm using a SpectraMax iD3 multi-mode microplate reader (Molecular Devices, San Jose, CA, USA).

### 2.4. Quantitative RT-PCR Analysis

Total RNA was extracted from hepatic and intestinal tissues using Trizol reagent (Takara Bio Inc., Kusatsu, Japan). cDNA was synthesized using a reverse transcription kit (PrimeScript RT Master Mix, Takara Bio Inc., Kusatsu, Japan). Quantitative PCR was performed on an Eppendorf Master cycler (Eppendorf SE, Hamburg, Germany) using SYBR Green Master Mix (Bio-Rad Laboratories, Inc., Hercules, CA, USA). Target gene expression was normalized to 18S using the 2^−ΔΔCt^ method [11]. Primer sequences for *Apob*, *Mttp* and other targets are listed in Table 1.

### 2.5. Histological Analysis

For lesion assessment, the heart and entire aorta were perfused with PBS and fixed in 4% paraformaldehyde.

En Face Analysis: The full-length aorta was opened longitudinally and stained with Oil Red O to visualize lipid-rich plaques.

Aortic Root Sections: The aortic roots were embedded in OCT compound (for Oil Red O staining) or paraffin (for H & E staining). Serial sections (8 μm thick) were prepared and stained to quantify plaque area and necrotic core size. Images were captured using a light microscope and analyzed with ImageJ software (version 1.53t, NIH, Bethesda, MD, USA).

Mouse liver, inguinal white adipose tissue, epididymal white adipose tissue, and interscapular brown adipose tissue were harvested and fixed in 10% neutral buffered formalin solution (Sigma-Aldrich Corp., St. Louis, MO, USA) and embedded in paraffin. The 5 μm-thick tissue sections were deparaffinized, rehydrated, and stained with hematoxylin and eosin (H & E).

### 2.6. Immunoblotting Analysis

Liver and intestinal tissues were homogenized in RIPA lysis buffer supplemented with protease and phosphatase inhibitor cocktails. Protein concentration was determined using a BCA Protein Assay Kit (Thermo Fisher Scientific Inc., Waltham, MA, USA). Equal amounts of protein (30 μg) were separated by SDS-PAGE and transferred onto 0.45 μm NC membranes (EMD Millipore Corp., Billerica, MA, USA). After blocking with 5% non-fat milk, membranes were incubated overnight at 4 °C with primary antibodies (1:1000 dilution). Following incubation with HRP-conjugated secondary antibodies, protein bands were visualized using an ChemiDoc XRS+ Imaging System (Bio-Rad Laboratories, Inc., Hercules, CA, USA). Band intensity was quantified using ImageJ software and normalized to β-Actin.

### 2.7. Cell Culture and Treatment

HepG2 and Hepa1-6 cell lines were obtained from the American Type Culture Collection (ATCC). Cells were cultured in DMEM supplemented with 10% fetal bovine serum (FBS, Procell Life Science & Technology Co., Ltd., Wuhan, China) and 1% penicillin/streptomycin at 37 °C in a humidified atmosphere containing 5% CO_2_. To induce hepatic steatosis, cells were incubated with 300 μM oleic acid (OA) conjugated with fatty acid-free BSA for 24 h. For GM3 treatment, cells were co-incubated with OA and GM3 (10 μM or indicated concentrations) for 24 h. Control cells were treated with BSA vehicle only.

### 2.8. Cell Viability Assay

Cell viability was determined using the MTT assay. For toxicity testing, cells were seeded in 96-well plates at a density of 1 × 10^4^ cells per well and cultured until they reached approximately 90% confluence. The cells were then treated as described above. After treatment, 20 μL of MTT solution (5 mg/mL) was added to each well and incubated for 4 h. The formed formazan crystals were dissolved in DMSO, and the absorbance was measured at 570 nm using a microplate reader.

### 2.9. Oil Red O Staining (In Vitro)

Cells cultured in 12-well plates were washed with PBS and fixed with 4% paraformaldehyde for 30 min. After fixation, cells were stained with Oil Red O working solution for 15 min at room temperature. For quantitative analysis, the dye was extracted with 100% isopropanol, and the absorbance was measured at 510 nm.

### 2.10. Statistical Analysis

During the conduct of the animal experiments, the investigators were aware of the group allocations. However, to minimize potential bias, the outcome assessments (including the histological quantification of atherosclerotic plaques and biochemical analyses) and subsequent data analyses were performed by investigators who were blinded to the group allocations.

All values are expressed as the mean  ±  standard error of mean, and analyses were performed using GraphPad Prism 10.0 (GraphPad Software, Boston, MA, USA). Data normality was assessed using the Shapiro–Wilk test. For normally distributed data, statistical significance was determined using Student’s *t*-test (two groups) or one-way ANOVA followed by Tukey’s post hoc test (multiple groups). For non-normally distributed data (e.g., ALT/AST ratios), non-parametric tests (Mann–Whitney U test or Kruskal–Wallis test) were performed. *p* < 0.05 was considered statistically significant.

## 3. Results

### 3.1. Exogenous GM3 Attenuates Plaque Progression and Ameliorates Hyperlipidemia

The ApoE^−/−^ mouse is a well-established model for studying atherosclerosis (AS) [12]. When fed a high-fat diet, these mice develop severe hyperlipidemia and atherosclerosis. In the aortic arch, the shear stress of blood flow alters endothelial cell morphology and increases the permeability of macromolecules such as LDL, making this region particularly susceptible to the formation of atherosclerotic plaques [13].

**Figure 1:**
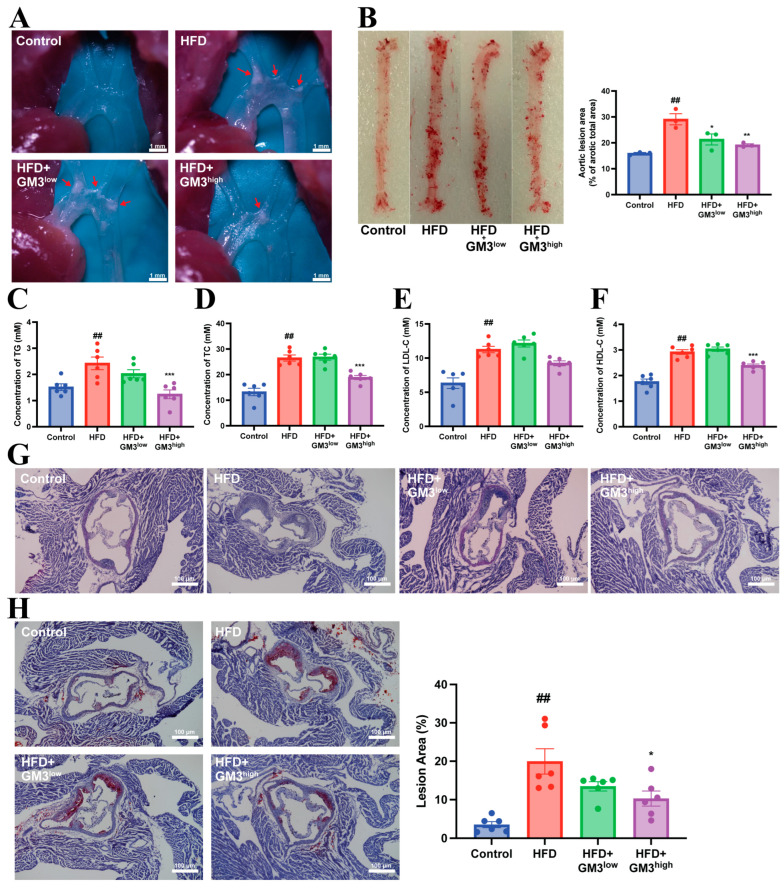
Exogenous GM3 inhibits the development of atherosclerosis in HFD-fed ApoE^−/−^ mice. (**A**) Representative Aortic arch (red arrows indicate atherosclerotic plaques) (scale bar: 1 mm). (**B**) Representative Oil Red O staining of full-length aorta (including the aortic arch, thoracic aorta, and abdominal aorta) and corresponding quantification of atherosclerotic lesions (*n* = 3). (**C**–**F**) Lipid profiles in serum (from left to right): triglyceride (TG), total cholesterol (TC), LDL-cholesterol (LDL-C), and HDL-cholesterol (HDL-C), respectively (*n* = 6). (**G**) Representative hematoxylin and eosin (H & E) staining of aortic root sections (scale bar: 100 μm). (**H**) Representative Oil Red O staining of aortic root sections and corresponding quantitative analysis (*n* = 6, scale bar: 100 μm). Data were expressed as means ± S.E.M of six mice in each group. ^##^ *p* < 0.01 vs. Control group; * *p* < 0.05, ** *p* < 0.01 and *** *p* < 0.001 vs. HFD group.

To evaluate the therapeutic impact of GM3 on atherosclerosis, we administered GM3 to ApoE^−/−^ mice at two dosages (1 and 4 mg/kg/3 days). Morphological analysis of the aorta using Oil Red O staining revealed that GM3 treatment significantly restricted lesion development. In the high-dose group (GM3^high^), plaque areas in the aortic arch were markedly reduced compared to the model group (Figure 1A). Similarly, en face analysis of the full-length aorta confirmed a significant reduction in total atherosclerotic lesion area (Figure 1B). Histological examination of the aortic root further supported these findings, with H&E and Oil Red O staining showing decreased plaque size and lipid burden in GM3-treated mice (Figure 1G,H).

Parallel to the reduction in plaque burden, GM3 treatment led to a dose-dependent improvement in systemic lipid profiles. While the low-dose treatment primarily affected triglyceride (TG) levels (Figure 1C), the 4 mg/kg dose effectively lowered total cholesterol (TC) and LDL-C as well (Figure 1D,E). Notably, plasma HDL-C levels remained comparable between the HFD and GM3-treated groups (Figure 1F), indicating that the hypolipidemic effect of GM3 is selective for pro-atherogenic lipoproteins. These findings demonstrate that exogenous GM3 provides potent protection against arterial plaque formation by correcting hyperlipidemia.

### 3.2. Hepatic Lipid Redistribution

**Figure 2:**
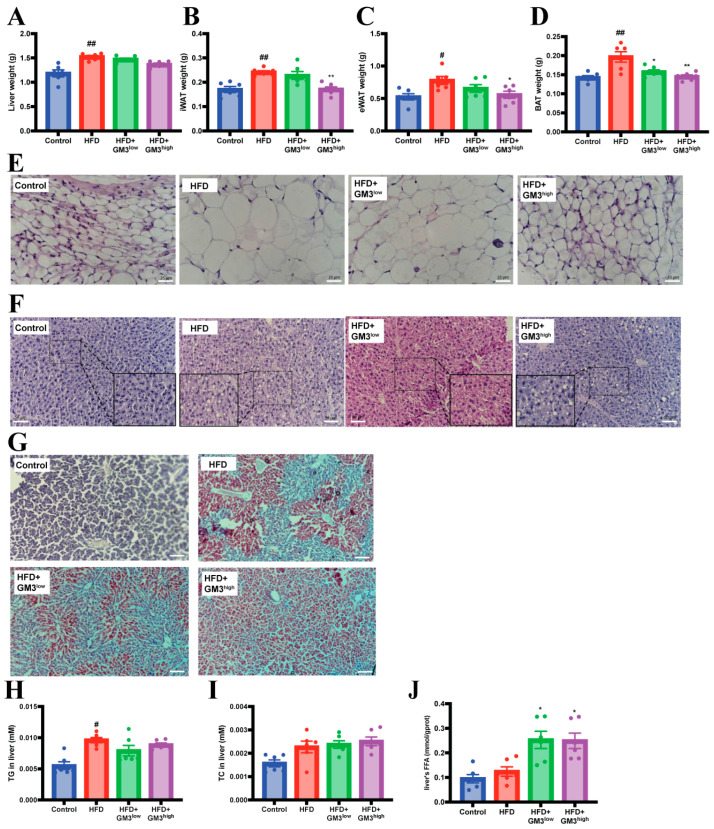
Effects of exogenous GM3 on the liver and adipose tissue in HFD-fed ApoE^−/−^ mice. (**A**–**D**) Liver, iWAT, eWAT and BAT weight (*n* = 6). (**E**) Representative H & E staining of inguinal white adipose tissue (scale bar: 25 μm). (**F**) Representative hematoxylin and eosin (H & E) staining of liver sections (scale bar: 50 μm). (**G**) Representative Oil Red O staining of liver sections (scale bar: 100 μm). (**H**–**J**) TG, TC and FFA in liver. ^#^ *p* < 0.05, ^##^ *p* < 0.01 vs. Control group; * *p* < 0.05 and ** *p* < 0.01 vs. HFD group.

To evaluate potential systemic effects, organ weights were recorded at the experimental endpoint. As shown in Figure 2A–D, the weights of the liver, iWAT, eWAT, and BAT remained unaltered across all treatment groups. Consistent with the macroscopic observations, H&E staining of iWAT sections revealed no obvious morphological abnormalities or adipocyte hypertrophy (Figure 2E), indicating that GM3 did not induce organ hypertrophy or atrophy. Interestingly, despite the reduction in circulating lipids, histological examination via H&E and Oil Red O staining revealed increased hepatic vacuolization and lipid droplet accumulation in the GM3-treated mice relative to the HFD group (Figure 2F,G). This observation was corroborated by elevated hepatic levels of triglycerides, total cholesterol, and free fatty acids (Figure 2H–J). Together, these findings suggest a lipid redistribution effect, wherein lipids are sequestered in the liver rather than retained in the systemic circulation.

### 3.3. GM3 Suppresses Hepatic VLDL Assembly and Secretion

Given a trend of a reduction in circulating LDL-C and triglycerides observed in the high-dose GM3 group, we hypothesized that GM3 acts upstream to restrict the hepatic output of atherogenic particles. Since the liver is the primary site of lipoprotein production, we investigated the impact of GM3 on the VLDL assembly machinery.

**Figure 3:**
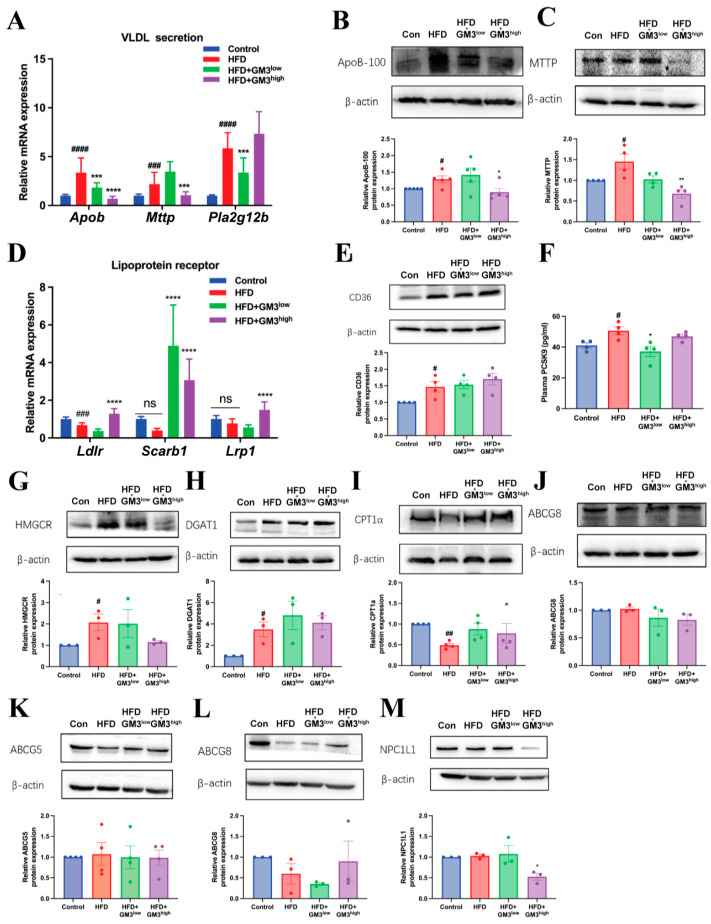
Exogenous GM3 modulates the expression of key genes and proteins involved in lipid metabolism in the liver and intestine. (**A**) Hepatic mRNA expression levels of *Apob*, *Mttp*, and *Pla2g12b* related to VLDL assembly. (**B**,**C**) Representative Western blots and quantitative analysis of ApoB100 (**B**) and MTTP (**C**) protein expression in the liver. (**D**) Hepatic mRNA expression levels of lipoprotein receptors (*Ldlr*, *Scarb1*, and *Lrp1*). (**E**) Protein expression of CD36. (**F**) PCSK9 level in plasma (*n* = 4). (**G**–**J**) Protein expression of HMGCR (**G**), DGAT1 (**H**), CPT1α (**I**) and ABCG8 (**J**) in the liver (*n* = 3). (**K**–**M**) Protein expression of ABCG5 (**K**), ABCG8 (**L**) and NPC1L1 (**M**) in the small intestine (*n* = 3). ns, not significant; ^#^ *p* < 0.05, ^##^ *p* < 0.01, ^###^ *p* < 0.001, ^####^ *p* < 0.0001 vs. Control group; * *p* < 0.05, ** *p* < 0.01, *** *p* < 0.001, and **** *p* < 0.0001 vs. HFD group.

We focused on Apolipoprotein B100 (*Apob*) and Microsomal Triglyceride Transfer Protein (*Mttp*), two rate-limiting factors essential for the lipidation and assembly of nascent VLDL particles. Quantitative RT-PCR analysis revealed that GM3 treatment significantly downregulated the hepatic mRNA expression of both *Apob* and *Mttp* in a dose-dependent manner (Figure 3A). Consistent with the transcriptional changes, immunoblotting analysis confirmed a marked reduction in hepatic ApoB100 and MTTP levels (Figure 3B,C).

Furthermore, we examined the expression of *Pla2g12b*, a phospholipase that plays a critical role in VLDL transport and secretion. As shown in Figure 3A, GM3 treatment significantly suppressed *Pla2g12b* mRNA levels compared to the HFD group. Collectively, these findings demonstrate that GM3 attenuates hyperlipidemia by inhibiting the molecular machinery required for hepatic VLDL assembly and secretion.

### 3.4. Transcriptional Upregulation of Hepatic Lipoprotein Clearance Receptors

Beyond reducing lipid secretion, we examined whether GM3 also accelerates the removal of lipoproteins from the circulation. GM3 treatment elicited a significant upregulation in the mRNA expression of key hepatic receptors, including *Ldlr*, *Scarb1*, and *Lrp1* (Figure 3D). Interestingly, this upregulation was accompanied by a notable decrease in plasma PCSK9 levels (Figure 3F), a protein known to mediate the degradation of LDLR. These data imply a dual action for GM3 in the liver: it not only “shuts the gate” on VLDL secretion but also “clears the road” by enhancing receptor-mediated uptake of circulating lipids.

To comprehensively dissect the mechanism of hepatic lipid accumulation, we also evaluated other key metabolic pathways. Interestingly, the protein expression levels of CD36 (fatty acid uptake), HMGCR (cholesterol synthesis), DGAT1 (triglyceride synthesis), and CPT1α (fatty acid oxidation) remained largely unchanged (Figure 3G–J). Additionally, the hepatic efflux transporter ABCG8 showed no significant alteration (Figure 3K). These data suggest that the lipid-modulating effects of GM3 are specifically mediated through VLDL assembly and lipoprotein receptor pathways rather than general metabolic reprogramming. While these transcriptional changes are promising and strongly correlate with the observed decrease in plasma PCSK9, further validation at the protein level is warranted to fully confirm the functional enhancement of these clearance receptors.

### 3.5. Reduction in Intestinal Cholesterol Absorption via NPC1L1 Downregulation

To determine if the lipid-lowering effect was also mediated through the digestive system, we analyzed cholesterol transport in the small intestine. Specifically, we measured the expression of NPC1L1, the primary transporter responsible for intestinal cholesterol absorption. In mice treated with GM3, we observed a significant, specific downregulation of NPC1L1 protein expression in the jejunum (Figure 3M). While we observed no significant changes in the expression of the intestinal efflux transporters ABCG5 and ABCG8 (Figure 3K,L), the selective suppression of NPC1L1 suggests that GM3 restricts the exogenous supply of cholesterol, complementing its hepatic actions to achieve systemic lipid control.

### 3.6. Toxicity Assessment of Exogenous GM3

**Figure 4:**
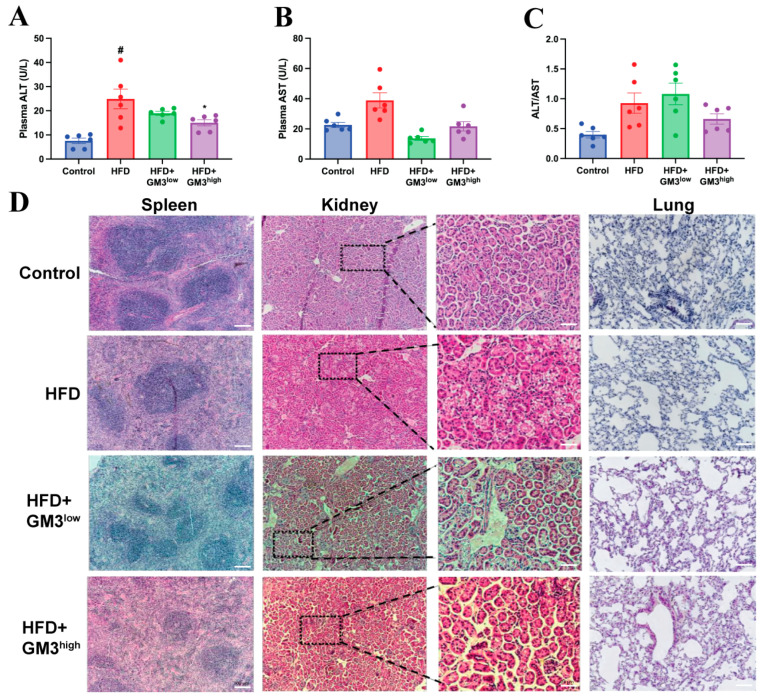
Effect of exogenous GM3 on high-fat diet-induced organ toxicity. (**A**–**C**) Plasma levels and ratio of ALT and AST (*n* = 6). (**D**) Representative hematoxylin and eosin (H & E) staining of spleen, kidney, and lung sections (scale bar: 100 μm). ^#^ *p* < 0.05 vs. Control group. * *p* < 0.05 vs. HFD group.

Despite this accumulation, the treatment did not induce hepatic injury. In fact, plasma levels of ALT and AST were significantly lower in the GM3-treated groups compared to the HFD model group (Figure 4A,B), and the ALT/AST ratio remained stable (Figure 4C). Furthermore, H&E staining of other major organs—including the spleen, kidney, and lung—showed no morphological abnormalities (Figure 4D). Combined with the preserved adipose tissue morphology observed earlier (Figure 2I), these results confirm that the therapeutic dosage of GM3 is well-tolerated and lacks systemic toxicity despite the observed hepatic lipid redistribution.

### 3.7. GM3 Facilitates Non-Toxic Lipid Accumulation in Hepatocytes

**Figure 5:**
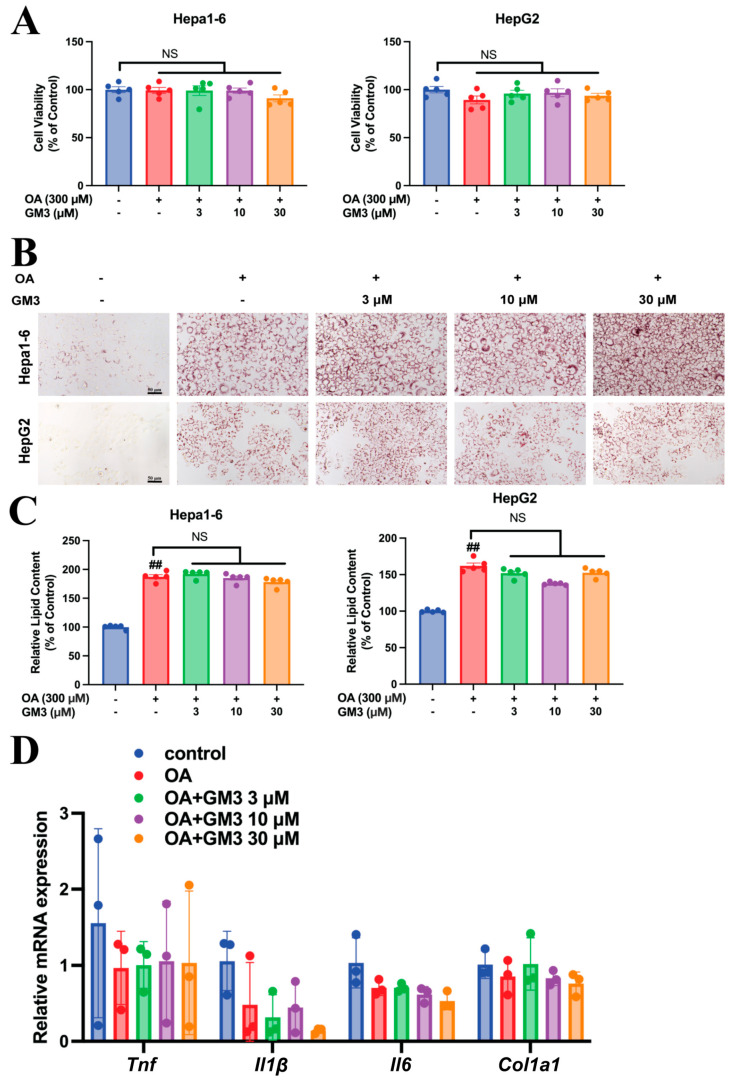
GM3 promotes safe lipid sequestration in hepatocytes in vitro. (**A**) Cell viability of Hepa1-6 and HepG2 cells treated with OA (300 μM) and GM3 for 48 h, assessed by MTT assay (*n* = 5). Data are expressed as percentage of the BSA control. (**B**) Representative Oil Red O staining images and quantitative analysis of lipid content in Hepa1-6 and HepG2 cells (*n* = 3, Scale bar: 50 μm). Lipid content was quantified by isopropanol extraction and optical density (OD) measurement at 510 nm. (**C**) Quantitative analysis of intracellular lipid content in Hepa1-6 and HepG2 cells. Lipid accumulation was quantified by isopropanol extraction and optical density (OD) measurement at 510 nm. (**D**) Relative mRNA expression levels of pro-inflammatory cytokines (*Tnf*, *Il1β*, *Il6*) and fibrotic marker *Col1a1* in Hepa1-6 cells were determined by RT-qPCR. Data are presented as mean ± SEM of at least three independent experiments. NS, not significant vs. OA model group. ^##^ *p* < 0.01 vs. OA model group.

To further validate the safety of hepatic lipid redistribution observed in vivo, we established an in vitro steatosis model using HepG2 and Hepa1-6 cells challenged with oleic acid (OA). First, to rule out potential cytotoxicity, cell viability was assessed using the MTT assay. As shown in Figure 5A, neither OA induction nor GM3 treatment (at various concentrations) impaired cell viability compared to the BSA control, indicating that the lipid loading was well-tolerated.

Next, quantitative analysis of Oil Red O staining confirmed significant lipid accumulation in both cell lines upon OA treatment. Notably, in Hepa1-6 cells, GM3 treatment maintained high intracellular lipid content like the OA model group (Figure 5B,C), mirroring the in vivo phenotype of hepatic lipid retention. Crucially, despite this substantial lipid burden, qPCR analysis revealed no significant elevation in pro-inflammatory markers (*Tnf*, *Il1β*, *Il6*) (Figure 5D). Furthermore, the expression of the fibro genic marker Col1a1 remained unchanged across all groups, suggesting that no fibrotic signaling was initiated. Collectively, these in vitro data strongly support the hypothesis of “safe lipid sequestration,” demonstrating that GM3 enables hepatocytes to accommodate excess lipids in metabolically inert droplets without triggering lipotoxicity, inflammation, or fibrosis.

## 4. Discussion

Previous studies have established GM3 as a physiological membrane component [9], but its potential as an exogenously administered therapeutic agent remains underexplored. In this study, we investigated the pharmacological effects of exogenous GM3 on atherosclerosis and lipid metabolism in ApoE^−/−^ mice. Our findings demonstrate that intravenous administration of GM3 significantly reduced aortic plaque burden. Unlike conventional therapies that target a single pathway, our data suggest that this anti-atherosclerotic effect is primarily driven by a systemic reprogramming of lipid metabolism along the hepatic–intestinal axis. Specifically, GM3 functioned as a bioactive lipid modulator that simultaneously inhibited intestinal lipid absorption and altered hepatic lipid flux, ultimately reducing circulating atherogenic lipoproteins.

An intriguing observation in our study was the apparent redistribution of circulating lipids to the liver. The concurrent hepatic accumulation of free fatty acids and the lack of adipose hypertrophy suggest that this lipid redistribution may be partially driven by enhanced peripheral lipolysis. Given that gangliosides are known to modulate insulin receptor signaling in lipid rafts, we postulate that an influx of exogenous GM3 might locally attenuate insulin sensitivity in adipocytes. This could disinhibit lipolysis, thereby promoting a continuous efflux of adipocyte-derived fatty acids that are subsequently sequestered by the liver. Mechanistically, GM3 treatment upregulated hepatic uptake receptors while inhibiting the VLDL secretion machinery, inevitably leading to hepatic lipid retention. This suppression of hepatic lipid output is strongly supported by previous in vitro evidence from Choi et al., who demonstrated that exogenous GM3 directly decreases the secretion of ApoB-100, an indispensable structural component for VLDL assembly in liver cells [14]. Crucially, however, this increased hepatic lipid droplet content was not accompanied by hepatocellular injury or inflammation, as evidenced by normal liver morphology and reduced serum transaminase (ALT/AST) levels in vivo.

To rigorously validate this non-toxic phenotype, we conducted in vitro assays using hepatocytes loaded with 0.3 mM oleic acid (OA). While higher concentrations of OA (~1 mM) or the use of saturated fatty acids like palmitic acid are conventional stimuli for inducing lipotoxicity and cell death [15], we intentionally selected this moderate OA dosage to precisely recapitulate the simple steatosis observed in our in vivo model. This approach allowed us to simulate a state of high lipid burden without baseline apoptosis, thereby providing a robust platform to evaluate whether GM3 itself exacerbates lipid-induced stress. These experiments confirmed that despite high intracellular lipid content mimicking the in vivo state, cells maintained normal viability and showed no induction of pro-inflammatory (*Tnf*, *Il1β*, *Il6*) or fibrotic (*Col1a1*) markers. This profound lack of inflammatory response may be intrinsically linked to the chemical composition of the exogenous GM3 used in our study. Recent evidence highlights that the inflammatory properties of GM3 are highly dependent on its ceramide fatty acid chain length; species containing very long-chain fatty acids (VLCFA) tend to elicit pro-inflammatory responses, whereas those with long-chain fatty acids (LCFA) exhibit anti-inflammatory effects [16]. Given that approximately 90% of the fatty acid moiety in our GM3 preparation is stearic acid (C18:0, an LCFA), its structural nature inherently favors an anti-inflammatory microenvironment. This structural specificity provides a critical molecular basis for why our GM3 administration effectively partitions lipids without triggering secondary lipotoxicity. This distinguishes the observed phenotype from pathological non-alcoholic steatohepatitis (NASH) [17,18,19]. Unlike therapeutic strategies that aim to reduce hepatic steatosis to ameliorate metabolic dysfunction [20], we propose that GM3 promotes a strategy of “metabolic partitioning”, where excess circulating lipids are diverted into inert hepatic lipid droplets. This redistribution effectively uncouples hepatic steatosis from lipotoxicity, suggesting that GM3 improves systemic lipid tolerance by clearing atherogenic lipids from the circulation without compromising hepatocyte viability [21,22].

**Figure 6:**
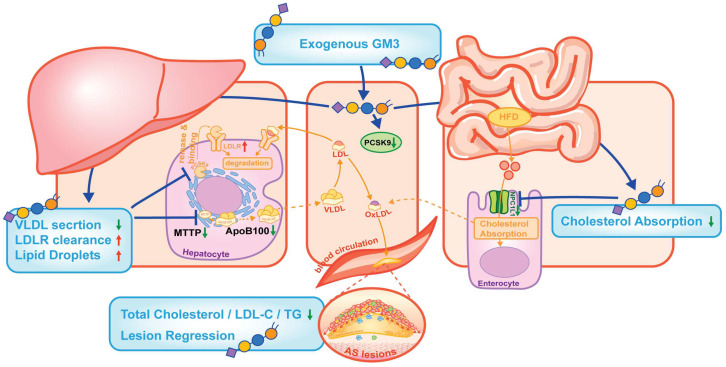
The multi-organ mechanisms of exogenous GM3 attenuate atherosclerosis. Exogenous GM3 exerts a comprehensive regulation on systemic lipid metabolism through three distinct pathways: (1) Hepatic “Dual-Hit”: GM3 suppresses VLDL assembly by downregulating ApoB100 and MTTP, while simultaneously enhancing LDLR-mediated clearance via the suppression of PCSK9. (2) Intestinal Blockade: GM3 restricts exogenous cholesterol absorption by downregulating NPC1L1 in the jejunum. These actions lead to a profound reduction in circulating lipids and the regression of atherosclerotic plaques.

Distinct from the local vascular effects observed in our previous studies (e.g., inhibition of LDL oxidation and monocyte adhesion), the current findings delineate the molecular mechanisms driving GM3’s systemic efficacy. As illustrated in Figure 6, GM3 exerts a “dual-hit” effect on hepatic lipid flux: it suppresses the assembly of atherogenic lipoproteins by downregulating ApoB100 and MTTP [23,24], while simultaneously enhancing receptor-mediated clearance via the suppression of plasma PCSK9 and the consequent transcriptional upregulation of *Ldlr* [25,26]. While our functional data clearly demonstrate a systemic reduction in circulating PCSK9, it remains to be elucidated whether this reduction is primarily driven by decreased hepatic synthesis (e.g., via transcriptional suppression of the Pcsk9 gene) or accelerated clearance. Although the precise upstream signaling warrants further mapping, gangliosides are known to modulate membrane lipid raft receptor tyrosine kinase (RTK) signaling—a mechanism we recently highlighted in the context of endothelial regulation and angiogenesis [27], potentially influencing nuclear transcription factors like SREBPs or HNF4α to drive these changes [28,29]. Importantly, this regulatory network extends to the intestine, where GM3 downregulates NPC1L1 in the jejunum—the primary target of ezetimibe—thereby restricting cholesterol influx [30,31]. Interestingly, a previous study demonstrated that endogenous GM3 in membrane lipid rafts is fundamentally required for NPC1L1-mediated cholesterol endocytosis [32]. While basal GM3 facilitates NPC1L1 activity, our findings reveal a novel pharmacological dimension: exogenous GM3 administration significantly reduces overall NPC1L1 protein levels. We postulate that an influx of exogenous GM3 may alter the precise stoichiometry of intestinal lipid rafts, potentially triggering the excessive internalization and subsequent degradation of NPC1L1, or serving as a negative feedback signal to restrict systemic cholesterol overload. In a similar vein, it is crucial to reconcile our systemic findings with previous literature demonstrating that GM3 synthase deficiency attenuates hypercholesterolemia [32], which might intuitively suggest that increasing GM3 bioavailability could be detrimental. We propose that this apparent paradox reflects the fundamental physiological difference between lifelong genetic ablation and adult-onset pharmacological intervention. A constitutive knockout of GM3 alters baseline metabolic wiring and systemic insulin sensitivity from birth. In contrast, our study employs a pharmacological bolus of exogenous GM3 in adult mice with established dyslipidemia. Rather than altering constitutive developmental architecture, this exogenous administration acts acutely to trigger systemic metabolic partitioning—actively redistributing atherogenic lipids from the circulation into the liver and downregulating lipid synthesis/absorption machineries. This underscores the dual nature of GM3: functioning as a basal structural requisite endogenously, while acting as a potent bioactive lipid modulator when administered exogenously.

Several limitations of this study should be acknowledged. First, while our conclusions emphasize systemic lipid-lowering and macroscopic plaque reduction, we did not comprehensively profile systemic inflammatory cytokines in the plasma. The direct effects of GM3 on the vascular wall remain to be fully characterized. Because GM3 can accumulate directly within atherosclerotic plaques, future studies should conduct targeted experiments on the aorta to explicitly evaluate its influence on endothelial function and local inflammation, and the microenvironmental regulation of lipoprotein–receptor interactions [33], specifically focusing on the roles of adhesion molecules (e.g., VCAM-1 and ICAM-1) and macrophage polarization markers. Second, regarding the safe lipid sequestration phenotype, the potential risk of liver lipotoxicity must be thoroughly considered. Although no inflammation or fibrosis was observed over the 12-week regimen, the persistent hepatic lipid burden carries a risk of progression to metabolic dysfunction-associated steatohepatitis (MASH) under chronic conditions. To rigorously rule out late-onset lipotoxicity, future research must involve long-term administration (e.g., 24–36 weeks) coupled with comprehensive fibrosis assessments, such as Sirius Red and Collagen I/III staining. Third, although body weight profiles were comparable between groups (which argues against significant caloric restriction), food intake was not strictly monitored quantitatively, preventing us from fully excluding subtle dietary effects. Finally, detailed pharmacokinetic data regarding exogenous GM3—specifically exact plasma concentrations, tissue biodistribution, and cellular uptake proportions—are lacking. Future investigations employing mass spectrometry-based lipidomics, isotope-labeled tracing, and nano-formulation strategies are warranted to accurately map its in vivo metabolic fate and optimize bioavailability. Nevertheless, by coordinating the blockade of hepatic synthesis, the promotion of clearance, and the restriction of intestinal absorption, GM3 offers a holistic advantage over agents that solely target synthesis and often trigger compensatory absorption [8,34].

## 5. Conclusions

Our study characterizes exogenous GM3 as a potent regulator of lipid metabolism. By restricting intestinal lipid absorption and redirecting circulating lipids into hepatic storage without inducing toxicity, GM3 effectively reduces the systemic atherogenic burden. Importantly, this multi-organ coordination highlights GM3’s potential as a multi-target therapeutic agent, offering a holistic advantage over conventional single-pathway treatments. These findings provide a compelling pharmacological rationale for developing GM3-based therapeutics for atherosclerosis and complex dyslipidemia.

## Figures and Tables

**Table 1:** RT-qPCR primers used in the study.

Target mRNA	Forward 5′-3′	Reverse 5′-3′
*Apob*	GCCCATTGTGGACAAGTTGATC	CCAGGACTTGGAGGTCTTGGA
*Mttp*	CAAGCTCACGTACTCCACTGAAG	TCATCATCACCATCAGGATTCCT
*P* *la2g12b*	CCAGCAATGACCAAGTGTTG	GAGAATCACAGGCTGCTGCTTCC
*L* *dlr*	TGACTCAGACGAACAAGGCTG	ATCTAGGCAATCTCGGTCTCC
*S* *carb1*	GGAGCATTCCTTGTTCCTA	TGCCCTTGACAGATTTGAT
*L* *rp1*	ACTATGGATGCCCCTAAAACTTG	GCAATCTCTTTCACCGTCACA
*Col1a1*	AATGTGGTTCGTGACCGTGA	AGCCTTGGTTGGGGTCAATC
*T* *nf*	TCAGCCTCTTCTCATTCCTG	CAGGCTTGTCACTCGAATTT
*I* *l1b*	CTGTGACTCATGGGATGATGATG	CGGAGCCTGTAGTGCAGTTG
*Il6*	CCTCTCTGCAAGAGACTTCCA	AGAATTGCCATTGCACAACTCT
*Rn18s*	TAAGTCCCTGCCCTTTGTACACA	GATCCGAGGGCCTCACTAAAC

## Data Availability

The original contributions presented in this study are included in the article. Further inquiries can be directed to the corresponding author.
